# Associations between childbirth, gang exposure and substance use among young women in Cape Town, South Africa

**DOI:** 10.1186/s13011-024-00610-0

**Published:** 2024-08-10

**Authors:** Felicia A. Browne, Tara Carney, Bronwyn Myers, Courtney Peasant Bonner, Wendee M. Wechsberg

**Affiliations:** 1 Substance Use, Gender, and Applied Research Program, RTI International, 3040 E. Cornwallis Road, Research Triangle Park, NC 27709 USA; 2 Health Behavior, University of North Carolina Gillings School of Global Public Health, 135 Dauer Drive, Chapel Hill, NC 27599 USA; 3 Alcohol, Tobacco and Other Drug Abuse Research Unit, South African Medical Research Council, PO Box 19070, Tygerberg, 7505 South Africa; 4 Division of Addiction Psychiatry, Department of Psychiatry and Mental Health, University of Cape Town, Cape Town, South Africa; 5 Curtin enAble Institute, Faculty of Health Sciences, Curtin University, Perth, WA 6845 Australia; 6 Maternal and Child Health, University of North Carolina Gillings School of Global Public Health, 135 Dauer Drive, Chapel Hill, NC 27599 USA

**Keywords:** Motherhood, Marijuana, Methamphetamine, Methaqualone, Mandrax, Young women, Gangs

## Abstract

The prevalence and influence of gangs on adolescents and young adults remain a concern in Western Cape, South Africa—particularly as they have one of the largest gang presence. While less attention has been focused on young women, there is a need to elucidate the relationship between gang exposure and health behaviors, such substance use, in addition to understanding whether becoming a caregiver impacts this relationship. This study uses baseline data from 496 participants enrolled in a NIDA-funded R01 trial that recruited young women aged 16 to 19 who were out of school and reported recent alcohol or other drug use and sexual risk behavior. At enrollment, a risk behavior survey was administered, and urine drug screening was conducted. Multivariable logistic regression analyses were conducted to examine baseline associations between childbirth, a gang exposure index based on eight items, and positive drug screens of the most prevalent drugs in the Western Cape (marijuana, methaqualone, and methamphetamine). At enrollment, approximately 39% of the sample had a positive urine screen for marijuana, 17% for methaqualone, and 11% for methamphetamine. Additionally, 28% had ever given birth. While only 6% reported ever being a member of a gang, most reported exposure to gangs through their physical and social environments. For all three drugs, gang exposure was associated with statistically significantly higher odds of a positive screen. Every one-point increase in the gang exposure index was associated with a 31% increase in the odds of a positive marijuana screen (*p* < .001), a 26% increase for methaqualone (*p* = 0.005) and a 37% increase in the odds of a positive methamphetamine screen (*p* < .001). Ever given birth was associated with lower odds of marijuana use (adjusted odds ratio [AOR]: 0.63; 95% CI: 0.42–0.96), but it was not associated with methaqualone or methamphetamine use. The findings suggest that exposure to gangs through young women’s social and physical environment is positively associated with drug use. Childbirth was also protective for marijuana use, indicating there may be something unique about this type of drug, such as one’s ability to more easily stop use. Although very few young women reported gang membership, a majority reported some exposure, indicating the need to address how pervasive this exposure is and the potential risk.

## Background

The prevalence of gangs and their association with substance use is a major health and social issue in the Western Cape Province of South Africa [1–4]. While a lot of research has focused on young men, this intersection is of particular concern among young women who have left school without graduating because both substance use and gang exposure can be reasons for leaving school early—or consequences of leaving school early [5, 6]. Similar relationships have also been observed with pregnancy [7]. Gangs can be attractive to young people because of their access to resources and substances [8]. For young women, for example, involvement with gangs may provide financial support for them or their families, which is also a primary reason they leave school.

Whether someone is an actual member of a gang or exposed to gangs in their social and physical environments has been shown to matter for risk and protective behaviors [9]. One U.S.-based study found that being in a gang was not associated with pregnancy among young women; however, having a partner affiliated with a gang was associated with pregnancy [10]. Previous research in South Africa has shown that youth exposed to gangs or gang members have higher rates of psychological distress [11].

Another key factor is motherhood, with research indicating that motherhood during adolescence may result in a temporary reduction of or abstinence from substance use and a reduction of gang involvement and exposure [12]. One Swedish study with young adult women found that reductions in substance use occurred both during pregnancy as well as postpartum [13]. In South Africa, of the young women who leave school early, family obligations are the third most common reason for leaving (12%), after not being to afford fees (26%), and poor performance (22%); this isthe least common reason for young men leaving (0.1%) [14]. For some women who made the decision to leave school to be a caregiver, these major changes may also motivate other changes in the lives.

Given the prevalence of gangs in Cape Town, it is important to understand how gang exposure is associated with substance use for young women, while understanding the role of childbirth—a proxy for parenthood—in this relationship. This manuscript examines how gang exposure and childbirth are associated with substance use among young women who have left school, while accounting for both gang membership and other forms of gang exposure in one measure.

## Methods

### Cape Town Young Women’s Health CoOp

The Cape Town Young Women’s CoOp (CT YWHC) is a National Institute on Drug Abuse funded, cluster randomized trial that enrolled 500 young women aged 16 to 19 who had left school early and reported recent alcohol or other drug use and sexual risk behavior. Participants were recruited from 24 community clusters in the Cape Town metropolitan area.

At enrollment, an adapted risk behavior survey [15] that included sociodemographic information and a risk behavior assessment was administered via computer-assisted personal interviewing (CAPI) for the majority of questions and audio computer-assisted self-interviewing (ACASI) for more sensitive questions about topics such as violence. Participants also provided a urine sample for drug and pregnancy screening, and they were tested for HIV via a fingerstick. Benzodiazepines, cocaine, methamphetamine, ecstasy, opiates, and marijuana were tested for using a multi-panel test; methaqualone was tested using a one-test strip. This study was approved by the RTI International Institutional Review Board and the South African Medical Research Council’s Ethics Committee. Additional protocol details have been published previously [16].

### Measures

The outcomes of interest were positive drug screens for the most prevalent drugs of misuse in the Western Cape—marijuana, methaqualone, and methamphetamine—based on previous studies conducted by our study team with similar populations in this study area [17, 18].

The primary predictors of interest were gang exposure and ever given birth. Gang exposure was assessed via eight dichotomous questions that asked about membership and exposure via participants’ social and physical environment. These items were summed to create an index of gang exposure, with the total index score ranging from 0 to 8. Ever given birth served as a proxy for parenthood and was based on a dichotomous single-item measure assessing whether participants had ever given birth.

Additional covariates of interest included sociodemographic characteristics, such as population group (Black or Coloured—the terms used in South Africa), age (continuous), and education, which was recoded into a dichotomous variable based on the highest grade completed—to reflect completion of the compulsory level of education in South Africa (Grade 9 or above). While gangs are an issue in the Western Cape as a whole, some research has shown differences by population group with a higher proportion in predominately Coloured communities [2, 19].

### Analyses

Bivariate analyses were conducted to examine associations between baseline characteristics and childbirth. Multivariable logistic regression analyses were conducted to examine baseline associations between childbirth, gang exposure, and positive urine drug screens of the most prevalent drugs—marijuana, methaqualone, and methamphetamine. Age, population group and education were controlled for in all models.

Analyses were conducted in Stata MP/IC Version 17. The community cluster variable was accounted for in the standard errors in all logistic regression analyses, and *p*-values less than 0.05 were classified as statistically significant. A complete case analysis was conducted because less than 1% of the data were missing on any of the measures examined. The resulting analytic sample used for analyses was 496.

## Results

### Sample characteristics

Table 1 presents characteristics of the subset of 496 study participants at enrollment overall and by whether participants had ever given birth. A total of 197 participants (39.7%) had ever been pregnant (not shown) and 138 (27.8%) had ever given birth. At enrollment, approximately 38.5% of the sample had a positive urine screen for marijuana, 16.7% had a positive screen for methaqualone, and 10.5% a positive screen for methamphetamine. While 6.0% reported ever being a member of a gang, a large proportion of the sample reported exposure to gangs through their physical and social environments. The majority of the participants (87.7%) reported having gangs in their neighborhood or close to where they live, and nearly half (47.6%) reported having someone close to them killed by someone in a gang. The gang exposure index had a mean of 3.55 (SD = 1.90) [not shown].

**Table 1:** Baseline characteristics of subset of Cape Town Young Women’s Health CoOp participants (*n* = 496), by ever given birth

	Total (*N* = 496)		Ever given birth (*N* = 138)		Never given birth (*N* = 358)	
**Sociodemographic**						
Age, mean (SD)^***^	17.8	(1.1)	18.0	(0.9)	17.7	(1.1)
	N	%	N	%	N	%
**Population group**						
Black African	244	(49.2)	63	(45.7)	181	(50.6)
Coloured	252	(50.8)	75	(54.3)	177	(49.4)
**Completed compulsory (Grade 9+)**	346	(69.8)	250	(69.8)	96	(69.6)
**Employed/working**	43	(8.7)	12	(8.7)	31	(8.7)
Pregnancy test, positive^**^	56	(11.3)	7	(5.1)	49	(13.7)
**Drug screening tests**						
Marijuana, positive	191	(38.5)	44	(31.9)	147	(41.1)
Methaqualone, positive	83	(16.7)	27	(19.6)	56	(15.6)
Methamphetamine, positive	52	(10.5)	19	(13.8)	33	(9.2)
**Gang exposure**						
Gang members in neighborhood/close by	435	(87.7)	125	(90.6)	310	(86.6)
Ever had a boyfriend in gang	216	(43.5)	64	(46.4)	152	(42.5)
Had sex partners in past 6 months in gang or affiliated	81	(16.3)	21	(15.2)	60	(16.8)
Ever had a family member in gang	274	(55.2)	73	(52.9)	201	(56.1)
Ever member of gang	30	(6.0)	5	(3.6)	25	(7.0)
Hang out with anyone in a gang	178	(35.9)	42	(30.4)	136	(38.0)
Someone close hurt by gang	310	(62.5)	87	(63.0)	223	(62.3)
Someone close killed by gang	236	(47.6)	60	(43.5)	176	(49.2)

*SD* Standard deviation

^**^p < .01; ^***^p < .001

Only two statistically significant differences by childbirth were observed for the baseline characteristics, age, and having a positive pregnancy screening test at baseline. Participants who had ever given birth were more likely to be older and they were less likely to have a positive pregnancy screening test at baseline. Childbirth was not significantly associated with any of the gang exposure variables.

### Associations between positive drug screens, childbirth, and gang exposure

Table 2 presents the adjusted logistic regression models with ever given birth and the gang exposure index predicting positive drug screening tests. For all three drugs, the gang exposure index was associated with statistically significantly higher odds of a positive drug screen. For marijuana, every one-point increase in the gang exposure index was associated with an estimated 31% increase in odds of having a positive drug screen (*p* < .001). Similarly, a one-point increase in the gang exposure index was associated with approximately a 26% increase in odds of having a positive methaqualone test result (*p* = 0.005) and an estimated 37% increase in odds of having a positive methamphetamine screening result (*p* < .001).

Additionally for marijuana, ever given birth was independently associated with a 37% reduction in odds of having a positive marijuana screening result; this was not observed for the other methaqualone or methamphetamine.

**Table 2:** Adjusted models – associations between gang exposure index, ever given birth and positive drug screening tests, controlling for age, population group, and education

	Marijuana			Methaqualone			Methamphetamine		
	AOR	95% CI	*P*-value	AOR	95% CI	*P*-value	AOR	95% CI	*P*-value
Gang exposure index	**1.31**	**(1.18–1.46)**	**< .001**	**1.26**	**(1.07–1.47)**	**0.005**	**1.37**	**(1.21–1.55)**	**<.001**
Given birth *(ref = no)*	**0.63**	**(0.42–0.96)**	**0.030**	1.57	(0.83–2.95)	0.162	1.34	(0.69–2.62)	0.388
Age	1.09	(0.90–1.32)	0.391	1.23	(0.89–1.71)	0.210	1.17	(0.86–1.61)	0.318
Coloured *(ref = Black)*	**1.75**	**(1.09–2.81)**	**0.021**	0.55	(0.29–1.06)	0.072	0.81	(0.40–1.65)	0.563
Grade 9 + completed (ref = no)	**0.65**	**(0.43–0.97)**	**0.034**	**0.32**	**(0.16–0.65)**	**0.002**	**0.40**	**(0.25–0.64)**	**< .001**

*CI* Confidence internal, *AOR* Adjusted odds ratio

## Conclusions

Although few young women reported lifetime gang membership (6%), the majority reported multiple gang exposures, indicating the need to address how pervasive gangs are and the potential risk at this critical period of adolescence and young adulthood. Our study findings suggest gang exposure through young women’s social and physical environment is positively associated with the primary drugs assessed—marijuana, methamphetamine, and methaqualone. These findings support previous research in this region that has shown associations between gangs and substance use [3, 20].

One potential explanation is that marijuana has the longest half-life of the three drugs, which also may explain this study finding and may make it difficult to compare the drugs. Additionally, methaqualone is often used with marijuana, but with a shorter half-life, this use, may not be captured with the drug test.

Ever giving birth was associated with a 37% reduction in odds and appeared to be a protective factor for recent marijuana use. This finding is supported in the previous literature, with studies finding that young women may decrease or stop substance use during pregnancy and post-partum [12, 21]. Additionally, one U.S.-based study found that when young adults became parents, their marijuana use reduced and being the person primarily responsible for caregiving was also associated with reduced marijuana use [22]. These findings could be attributed to the awareness of the effects of marijuana, but it may also be a result of being motivated to make changes when they are assuming more responsibilities and may also be around others who are affected by this use. Additionally, it may be easier to reduce or stop marijuana use compared with methaqualone or methamphetamine, due to their strong physiological effects.

Assessing quantitively and qualitatively patterns of use and potential changes in use due to parenthood and other life stages will be key to understanding this mechanism. Elucidating this relationship will also provide opportunities for potential intervention, as there have been programs that have successfully reduced substance use among adolescent mothers, while increasing school retention and graduation and increasing social support [22].

This study has some limitations. First, the analyses use cross-sectional data and gang exposure measures did not assess magnitude or frequency of exposure as they were dichotomous. Second, the young women who were out of school were recruited for a community-based HIV risk-reduction trial, which may limit generalizability. Another limitation is that the self-reported data may have been subject to recall bias and social desirability bias as the majority of the survey—including the gang exposure questions—were collected via CAPI. Despite these limitations, the study has several strengths. First, the gang exposure index captured multiple forms of gang exposure—including membership—into one item. Second, the study used drug screening test data for the drug use outcomes, which is not subject to reporting bias. Additionally, there were limited missing data for these analyses. Lastly, with an estimated 40% of people in the Western Cape having not completed secondary school by 20, and most leaving school during the last few grades to complete—similar to what we observed among study sample—these findings may have relevance to a larger population.

The relationship between gangs, substance use, and parenthood is a notable health and social concern in this region of South Africa not only for young women in gangs, but those who are exposed to gangs. Adolescence is a critical developmental period where young women may be exposed to all three of these factors for the first time. Consequently, it is imperative that interventions consider the broader social determinants and contextual factors, such as resources and gender, to have the greatest impact with this key population of young women.

## Data Availability

The study dataset is available upon request submitted to the study Principal Investigator.

## Abbreviations

ACASI: Audio computer-assisted self-interviewing
AOR: Adjusted odds ratio
CAPI: Computer-assisted personal interviewing
CI: Confidence internal
CT YWHC: Cape Town Young Women’s CoOp
SD: Standard deviation
